# Long-term use of CPAP in patients with obstructive sleep apnea: a prospective longitudinal cohort study

**DOI:** 10.1007/s41105-021-00361-6

**Published:** 2021-12-18

**Authors:** Margareta Møkleby, Britt Øverland

**Affiliations:** 1 Institute of Health and Society, Medical Faculty, University of Oslo, Oslo, Norway; 2 Pediatric and Adult Sleep Disorder Clinic, Lovisenberg Diaconal Hospital, Oslo, Norway

**Keywords:** Obstructive sleep apnea, Continuous positive airway pressure, Long-term adherence

## Abstract

Continuous positive airway pressure (CPAP) is an efficient treatment for obstructive sleep apnea (OSA). Reports of long-term usage vary, as do the factors that predict long-term usage. The aim of this study was to explore long-term CPAP usage and identify potential predictors. This prospective longitudinal cohort study included all patients referred to an outpatient clinic for CPAP treatment during an eight-month period. Clinical data were collected at baseline. Follow-ups were scheduled after one week, three months and two years. Use data were downloaded from the CPAP device at each follow-up. Of 163 included patients, 112 were available for long-term follow-up 2–4 years after starting CPAP, and use data were downloaded for 99 patients. Median duration of CPAP use was 6 h/night (IQR 4.2–7.1). The only significant variable predicting long-term usage was usage at three months. Nearly half (43%) of the patients needed extra consultations beyond the standard treatment plan. Most patients (69%) did not contact the clinic for their recommended two-year follow-up but were instead called into the clinic specifically for the study. There was no significant difference in long-term CPAP usage between patients who initiated contact themselves and those who were called in and would otherwise have been lost for follow-up. Most patients adhere well to CPAP in the long term, although many need extra follow-up. Patients lost for follow-up should not necessarily be considered non-adherent as their reason for not attending could be that they are managing treatment well on their own.

## Introduction

Obstructive sleep apnea (OSA) is a disorder of breathing during sleep characterized by periodic obstruction of the upper airway that interferes with normal respiratory gas exchange and disturbs sleep. OSA is independently associated with daytime sleepiness, neurocognitive impairment, depression, cardiovascular disease, and all-cause mortality [1–4]. OSA is, in most cases, a chronic condition that needs lifelong treatment.

Continuous positive airway pressure (CPAP) is considered the first-line medical treatment for adults with moderate to severe OSA. CPAP has been shown to normalize sleep architecture, reduce daytime sleepiness, enhance daily function, elevate mood, reduce motor vehicle accidents, and decrease blood pressure and other cardiovascular events [5].

Optimal use of CPAP requires patient engagement, and a limitation of the treatment is that poor adherence is common. CPAP adherence rates reported in the literature vary considerably, with non-adherence ranging between 29 and 83% [6, 7] where the cut-off in most cases has been based on a standard of CPAP adherence for 4 h nightly [6, 8]. Most studies describe CPAP use in the short term (< 6 months after initial referral), and there are few studies reporting CPAP adherence rate in the long term (beyond 1 year) [9]. Many have tried to identify clinical factors that can predict long-term CPAP use, with conflicting results [6, 10, 11].

The aim of the present study was to investigate CPAP usage in the long term (2 years after initial referral) and to identify potential predictors to long-term adherence.

## Material and method

### Study population and design

This prospective longitudinal cohort study was conducted in a Norwegian outpatient sleep clinic. The patients were recruited consecutively during an eight-month period. The study enrolled patients who were at least 18 years old, able to communicate in Norwegian, diagnosed with OSA, and referred for CPAP treatment, but with no prior CPAP experience. All included patients provided informed consent, and the study was approved by the Norwegian Data Inspectorate (13/3003).

### Baseline clinical characteristics

Patient characteristics were collected at baseline, before initiation of CPAP treatment. To assess subjective daytime sleepiness, the Epworth Sleepiness Scale (ESS) was used, with scores ranging from 0 to 24 and scores of 11 or higher indicating excessive daytime sleepiness. Body mass index (BMI) was calculated as weight in kilograms divided by the square of height in meters. For most patients (97%), their diagnosis of OSA was determined by in-home polygraphy, while a few patients had in-hospital polysomnography. Sleep and respiration were scored according to the guidelines from the American Academy of Sleep Medicine. Sleep and respiration were scored according to the guidelines from the American Academy of Sleep Medicine [12]. The apnea–hypopnea index (AHI) was calculated based on the total number of events per hour of total recording time (polygraphy) or per hour of sleep (polysomnography). Marital status, education level and self-reported cardiovascular disease were assessed with questionnaires.

### Follow-up

The diagnosis of OSA and recommended treatment with CPAP were determined by an ENT-specialist. The recommendation for CPAP treatment was based on the severity of OSA and the patients´ symptoms. In cases with mild OSA where the patients perceived symptoms that greatly affected their daytime functioning, CPAP was tried out in order to evaluate the effect. There was no upper age limit concerning who could be prescribed a CPAP, and therefore all patients diagnosed with OSA that experienced symptoms were offered a CPAP trial. When needed, the interdisciplinary team made an overall assessment together with the patient of whether CPAP treatment could be carried out at all. Such cases could for instance be related to the patient´s life situation and cognitive and/or physical functioning.

Standard treatment procedure at the time of the study was an offer to participate in a voluntary nurse-led information course, followed by three individual consultations with a nurse.

The first consultation focused on providing information about the CPAP device and how to use it. The proper mask was chosen, and the patient tried out the equipment. Auto CPAP was used in all cases, but if needed, the CPAP pressure was adjusted. Based on the patients’ needs and preferences, a humidifier was also prescribed. All patients were followed up clinically after 1–2 weeks and again at three months, and data from the CPAP device were downloaded at both follow-up visits. The nurse and patient talked about how the treatment was working, and potential challenges related to CPAP use were addressed. Common solutions involved a change of mask, adjustments of the air pressure, review of maintenance procedures and discussion of strategies for adapting and adhering to the CPAP therapy. The patients could also contact a nurse over the phone on weekdays if they had questions and/or problems. Extra follow-up visits could be arranged if needed. Patients who managed with the three individual consultations scheduled were considered to have followed the standard treatment, whereas patients who needed consolations beyond this were not considered to have followed the standard treatment.

Follow-up visits were discontinued once downloaded CPAP data showed normalization of respiration, and the patient experienced improved sleep quality and/or beneficial subjective effects and managed the treatment on their own. No further follow-ups were scheduled, and the patients were told to contact the clinic after two years for a follow-up, or sooner if needed.

In Norway, patients receive a CPAP device along with the necessary equipment for free upon application from a medical specialist and, therefore, there was no charge for the study participants related to the CPAP treatment. Annually, or in case of damage, the patients could receive a new mask and tube at no cost from the Regional Health Authorities. At the time the study was conducted, the Regional Health Authorities required that all patients complete a trial period of CPAP before the ENT-specialist could apply for oral appliance therapy (OAT).

### Statistics

Statistical analyses were performed using SPSS version 24 (IBM Corp., Armonk NY). Differences in clinical characteristics between groups were assessed using the Mann–Whitney *U* test for non-normal continuous variables, and either Chi-square test or Fisher’s exact test for categorical variables. To assess whether there were any statistically significant associations between the outcome of long-term CPAP use and the selected covariates of age, gender, AHI, ESS > 10, BMI, cardiovascular disease, following standard treatment, and CPAP use at the 1-week and 3-month follow-up visits, we fitted a multiple linear regression model. Model fit was assessed using visual inspection and the normality of the distribution of residuals was assessed using histograms and Q-Q plots.

The results are expressed as estimates of beta with 95% confidence intervals (CI). *P*-values < 0.05 were considered statistically significant.

## Results

**Fig. 1:**
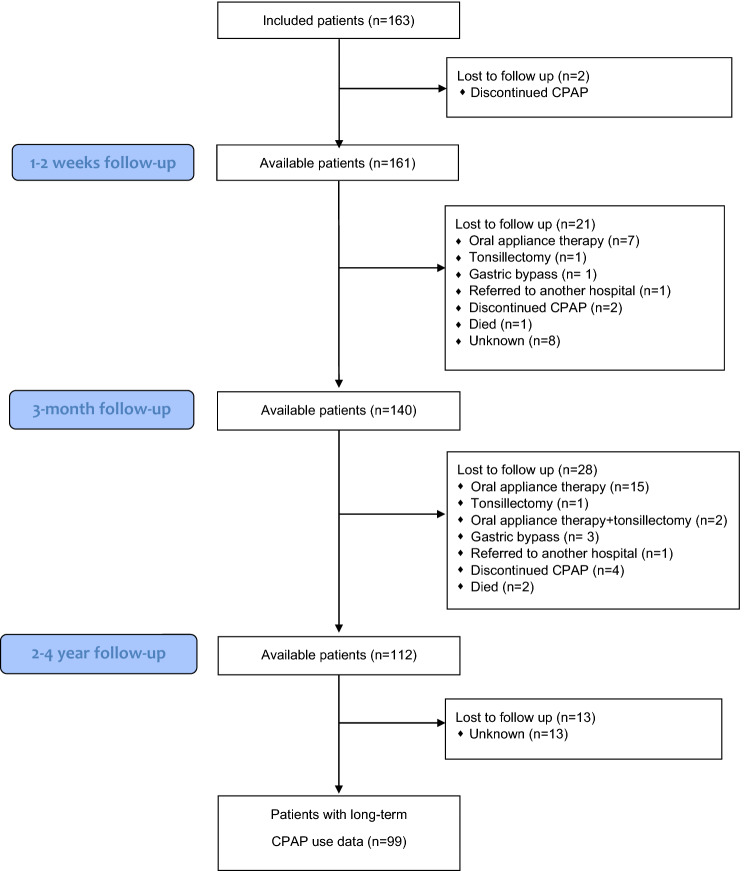
Participant flow chart

In the eight-month study period, CPAP treatment was initiated in 163 patients with various severities of OSA. The median age was 50 (range 21–79), median AHI 25,8 (range 1–139) and 20% were women. Two patients discontinued the CPAP treatment before the first follow-up due to severe anxiety and psychological problems. An additional 21 patients discontinued the CPAP treatment or were lost for follow-up before 3 months and 28 patients after the 3-month follow-up, resulting in 112 CPAP patients available for long-term follow-up. The reasons for discontinuing CPAP treatment were multifactorial: 47% were referred for treatment with OAT, while 16% of the patients did not attend the follow-up for an unknown reason. A participant flow chart is included as Fig. 1. The clinical characteristics of the patients available for long-term follow-up (*n* = 112), and those lost for follow-up (*n* = 51) are summarized in Table 1. The patients who continued CPAP use had a significant higher age and higher AHI than those who discontinued CPAP; we found no other significant differences in clinical characteristics between the two groups.

**Table 1:** Clinical characteristics at baseline for patients available for long-term follow up and lost to follow up

	Available for long-term follow up			Lost to follow up		
	*n*			*n*		*p*
Age; median (IQR)	112	51.5 (43.3–61.0)		51	46.0 (37.0–59.0)	0.04
Gender, female; *n* (%)	112	19 (17)		51	14 (28)	0.12
AHI; median (IQR)	112	30.0 (19.0–46.5)		51	21.0 (14.7–31.0)	< 0.01
ESS; median (IQR)	107	9 (5.0–12.0)		50	9.0 (6.0–14.0)	0.77
ESS ≥ 11; *n* (%)	107	41 (38)		50	18 (36)	0.78
BMI; median (IQR)	112	31.0 (26.9–33.0)		50	27.7 (25.0–33.0)	0.10
Cardiovascular disease; *n* (%)	111	48 (43)		50	17 (34)	0.27
Higher education; *n* (%)	110	58 (53)		50	24 (47)	0.50
Living in a relationship *n* (%)	112	79 (71)		50	29 (58)	0.12

*CPAP *continuous positive airway treatment;* IQR *interquartile range; *AHI *apnea–hypopnea index, the number of apneas and hypopneas per hour of sleep;* ESS *Epworth

Sleepiness Scale; *BMI *body mass index

Downloaded data were obtained from 99 of the 112 patients at the long-term follow-up at a median of 3 years (IQR 2.6–3.6). There were no significant differences in clinical characteristics between the 99 patients whose CPAP use data was downloaded, and the 13 patients lost for follow-up. The median duration of CPAP use was 6 h per night (IQR 4.2–7.1). Of the 99 patients, 78 (79%) used the CPAP > 4 h/night, and 51 (52%) used it > 6 h/night.

We performed a multivariate linear regression, to investigate whether any of the selected and potentially predictive factors were associated with long-term CPAP adherence (Table 2). The only predictor that was significantly associated with long-term CPAP adherence was CPAP use at 3 months.

**Table 2:** Multivariate linear regression predicting long-term CPAP use

	Unadjusted			Adjusted		
	B	95% CI	*p*	B	95% CI	*P*
Age	0.02	– 0.02 to 0.05	0.39	0.01	– 0.02 to 0.05	0.40
Gender	0.49	– 0.76 to 1.75	0.44	0.61	– 0.39 to 1.61	0.23
AHI	0.01	– 0.01 to 0.03	0.29	0.01	– 0.003 to 0.03	0.12
ESS > 10	-0.88	– 1.80 to 0.04	0.06	0.11	– 0.60 to 0.82	0.76
BMI	0.03	– 0.05 to 0.11	0.41	–0.02	– 0.09 to 0.05	0.60
Cardiovascular disease	– 0.73	– 1.63- 0.17	0.11	0.13	– 0.63—0.88	0.74
Standard treatment	– 0.38	– 1.28 to 0.53	0.41	0.25	– 0.44 to 0.94	0.48
CPAP-use (first follow-up)	0.32	0.08–0.55	0.01	– 0.15	– 0.38 to 0.08	0.19
CPAP-use (3-months follow-up)	0.88	0.72–1.03	< 0.001	0.94	0.73–1.16	< 0.001

*CPAP *continuous positive airway treatment,* CI *confidence interval,* AHI *apnea–hypopnea index, the number of apneas and hypopneas per hour of sleep,* ESS *Epworth Sleepiness Scale,* BMI *body mass index

**Fig. 2:**
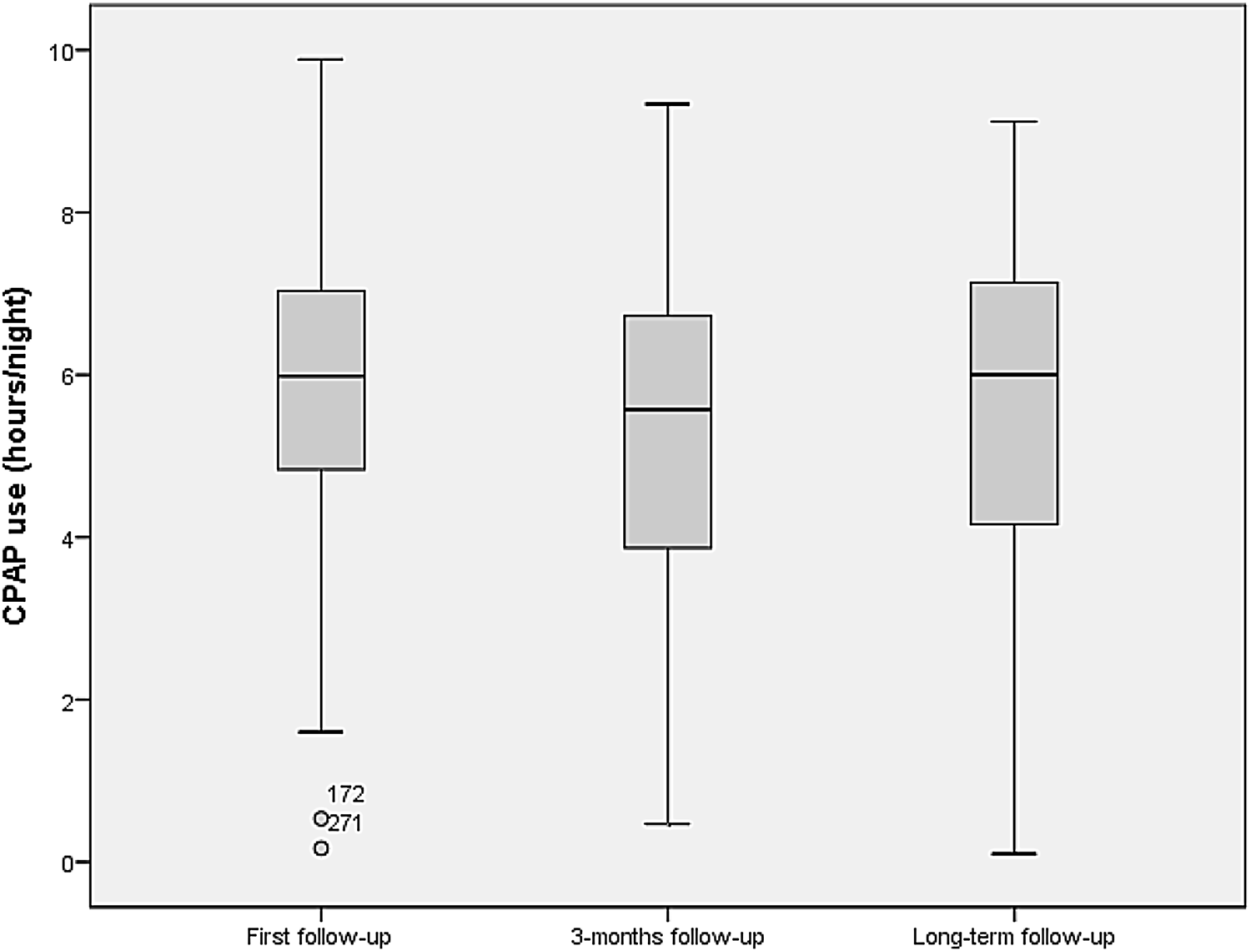
Distribution of CPAP use (hours/night) at each follow-up

Variation in CPAP use at each follow-up visit is illustrated in Fig. 2. The median duration of CPAP use was 6.0 h/night (IQR 4.8–7.0) at the first follow-up, 5.6 h/night (IQR 3.8–6.8) at the 3-month follow-up, and 6 h/night (IQR 4.2–7.1) at the long-term follow-up. There was a decline in use from the first follow-up to the 3-month follow-up (*p* = 0.001), but there was no significant change in use from the 3-month follow-up to the long-term follow-up (*p* = 0.98).

Only 20% of the patients initiated their own long-term follow-up visit, as recommended. Most patients (69%) were called in by the study nurse for the purpose of this study. The remaining 11% were followed at the clinic on a regular basis for various reasons. When comparing long-term CPAP use for patients who initiated their own follow-up visit (median use of 6.1 h/night, IQR 4.6–7.3) with patients who were invited by the study nurse (median use of 5.5 h/night, IQR 4.1–7.1), there was no significant difference (*p* = 0.28). In addition, there were no significant differences in clinical characteristics between the two groups.

Almost half (43%) of the patients needed extra consultations beyond the standard treatment plan. However, there was no difference in long-term CPAP use or clinical characteristics between the patients needing extra consultations and those following the standard treatment plan.

## Discussion

In this sample of patients, the majority (69%) were available for long-term follow-up. After a median time of 3 years, downloaded CPAP data indicated median use of 6 (IQR 4.2–7.1) hours per night. An important finding was that the patients who did not initiate their own long-term follow-up and were only assessed for the purpose of this study used CPAP on a regular basis with no significant difference in use when compared to patients who initiated their own long-term follow-up visit. The only significant predictor of long-term CPAP adherence was CPAP use at the 3-month follow-up. Most (57%) patients followed the standard treatment plan, but 43% needed extra appointments with a nurse or an ENT-specialist to achieve satisfactory CPAP use.

In the comparison of the group of patients who continued with CPAP and those who did not, the first population had higher AHI and higher age. The finding of higher AHI could imply that they had more symptoms and had no choice but to continue CPAP treatment even though this was not necessarily revealed in the ESS. Additionally, with higher AHI, the indication for an alternative treatment to CPAP was in many cases not an option. The reason why younger patients more often abandoned CPAP treatment than the elderly could be due to the fact that an oral appliance for younger patients was more often an alternative with regard to dental health and life situation.

CPAP adherence has been a focus for numerous studies [6, 9, 13–16]. However, Sawyer and colleagues (2011) raised a key question when asking *“Can CPAP adherence be accurately measured?”*. Adherence is often understood as hours of use per night, where the cut off and definition for being an adherent user varies. For long-term adherence, there is no agreement on which study population the measurements should be based on. Assessing adherence could, therefore, be based on all patients for whom CPAP therapy was recommended, or only those actually continuing CPAP use after the trial period. This inconsistency is reflected in the considerable variation in reported CPAP adherence [13]. When the criteria are vague for whom to consider a CPAP user, it is difficult both to define the non-users in the long-term but also to compare studies. This variance and differences come into sight in a systematic literature review on trends in CPAP adherence where papers identified for possible analysis on the subject consisted of six different categories on CPAP trials [9]. Even though such a systematic literature review on CPAP adherence has large varieties and there are many factors to adjust for, it nevertheless brings focus to how CPAP adherence is hard to completely define, measure and compare. For the present study, 69% of all patients were available for long-term follow-up, and data from the device were downloaded for the majority (99 out of 112 patients), showing a median duration of CPAP use of 6 h/night (IQR 4.2–7.1). This is similar to findings from other studies that report mean use of 5.5 ± 1.9 h at 3 years and median use of 5 h after 4 years [14, 15].

Of the 163 patients included in this study, 51 patients were not accessible for long-term follow-up (Fig. 1). The majority of these patients had switched to another treatment, such as oral appliance therapy or surgery. There was a low threshold for recommending CPAP therapy at the study clinic. Additionally, due to the guidelines from the Regional Health Authorities at the time of the study, all patients had to try a CPAP device before an oral appliance therapy could be prescribed.

Based on publications in the 1990s, where findings from several studies showed mean usage just above 4 h nightly, this unintentionally became a standard for CPAP adherence [6, 8]. More recent studies have observed that adherence for ≥ 6 h per night was associated with more significant improvements in daytime symptoms [6, 17]. In both research and clinical settings, the approach to CPAP adherence has mainly been binary, classifying patients as either adherent or non-adherent related to user time per night and per week, where patients who continually attempt to use CPAP but present with low user times have been counted as non-adherent [8]. In this study, we decided not to classify the patients as adherent or non-adherent based on their user time as there is no clear agreement on how to define adherence /non-adherence and not all patients easily fit the groups. Further, neither those who stopped using CPAP, nor those lost for follow-up are categorized as non-adherent as we argue this would give a skewed picture. This differs from other studies, where the groups of patients mentioned above have been labeled non-adherent [15].

When the routine follow-ups were completed at three months, the patients were told to contact the clinic after two years for a long-term follow-up visit. Most of the patients did not initiate contact with the clinic on their own, and two thirds of the patients were therefore called in specifically for the study. When these latter patients came to their long-term follow-up, downloaded data from their CPAP showed a median use of 5.5 h/night (IQR 4.1–7.1), which was not significantly different from the CPAP use of those who contacted the clinic on their own (median use of 6.1 h/night, IQR 4.6–7.3). The main reason patients reported for not contacting the clinic was that using CPAP had become a well-functioning everyday routine and they did not need further help or support from health care personnel. This result substantiates an assumption presented in an earlier study concerning long-term adherence, where patients who used CPAP for > 4 h per night at the most recent appointment most likely had a similar user profile in the long term, even if they were lost for follow-up [14]. We, therefore, consider the result from the present study as important. This shows how more patients than first presumed use CPAP in a long term. Additionally, such finding might motivate health care personnel in the job of helping and encouraging new patients to adapt to CPAP.

Adapting to CPAP and integrate the device into everyday life is a process, and it has been outlined in earlier publications that adherence is a complex multifaceted phenomenon, where interventions need to be personalized [8, 10, 13, 18–20]. Although the majority of patients in this study managed well on their own in the long run, more than 40% of the study population needed extra appointments at the clinic beyond the standard follow-up plan. The reasons for extra support could be related to adjusting the equipment, encouragement or further investigation of disturbed sleep. These findings highlight the varied challenges patients might face, the varied interventions health care personnel need to provide, and how it is not enough to prescribe a CPAP device and assume that the patient’s OSA is being adequately treated, as pointed out in earlier research [9].

For the present study, the only significant variable predicting long-term CPAP usage was user time at the three-month follow-up (*p* < 0.001). This finding is similar to those of other studies where early CPAP adherence measurements were a predictor of long-term adherence [16, 21–23]. Other factors, such as age, socioeconomic status, the severity of OSA and/or symptoms and social support, have been being associated with adherence in some studies, but the findings are inconsistent [6, 10, 11].

Being able to predict patients’ long-term adherence would be of great benefit, but as the device is used in the patient’s personal context, potential predictors nevertheless need to be assessed in each prevailing situation. Such an individual approach might be especially important in the early treatment period to lay the groundwork for sustainable long-term CPAP adherence.

## Limitations of the study

Participants were included from one single urban sleep center. However, the clinic had a large catchment area, which ensured a mix of patients from urban and rural areas. The study sample could have been larger, but reflects the pre-defined short inclusion period. The patients were included consecutively and were representative of the population at the study clinic, although generalizability may be limited.

In the present study, several nurses were involved in the CPAP follow-up visits and although they all followed the clinic’s guidelines, there was no specific study protocol and the approach and interaction with the patients may have varied.

## Conclusions

Most patients adhere well to CPAP in the long-term. The only predictor of long-term adherence is CPAP use 3 months after starting treatment. Many patients need more follow-up than scheduled in the standard treatment plan, and it is important that health care personnel are responsive to the individual challenges that may occur and have the option to offer extra support when needed.

Patients lost for follow-up should not automatically be considered non-adherent. The present study shows that the majority of patients did not contact the clinic for long-term follow-up and the most common reason was because CPAP use had become well integrated into their everyday life and further support was not needed.
